# Activity Detection in Indoor Environments Using Multiple 2D Lidars

**DOI:** 10.3390/s24020626

**Published:** 2024-01-18

**Authors:** Mondher Bouazizi, Alejandro Lorite Mora, Kevin Feghoul, Tomoaki Ohtsuki

**Affiliations:** 1 Faculty of Science and Technology, Keio University, Yokohama 223-8522, Japan; bouazizi@ohtsuki.ics.keio.ac.jp; 2 Graduate School of Science and Technology, Keio University, Yokohama 223-8522, Japan; alejandro@ohtsuki.ics.keio.ac.jp; 3 UMR-S1172—Lille Neuroscience and Cognition, Centre Hospitalier Universitaire Lille, Inserm, University of Lille, F-59000 Lille, France; kevin.feghoul@univ-lille.fr

**Keywords:** activity detection, human activity recognition, fall detection, healthcare, 2D Lidar, deep learning, machine learning

## Abstract

In health monitoring systems for the elderly, a crucial aspect is unobtrusively and continuously monitoring their activities to detect potentially hazardous incidents such as sudden falls as soon as they occur. However, the effectiveness of current non-contact sensor-based activity detection systems is limited by obstacles present in the environment. To overcome this limitation, a straightforward yet highly efficient approach involves utilizing multiple sensors that collaborate seamlessly. This paper proposes a method that leverages 2D Light Detection and Ranging (Lidar) technology for activity detection. Multiple 2D Lidars are positioned in an indoor environment with varying obstacles such as furniture, working cohesively to create a comprehensive representation of ongoing activities. The data from these Lidars is concatenated and transformed into a more interpretable format, resembling images. A convolutional Long Short-Term Memory (LSTM) Neural Network is then used to process these generated images to classify the activities. The proposed approach achieves high accuracy in three tasks: activity detection, fall detection, and unsteady gait detection. Specifically, it attains accuracies of 96.10%, 99.13%, and 93.13% for these tasks, respectively. This demonstrates the efficacy and promise of the method in effectively monitoring and identifying potentially hazardous events for the elderly through 2D Lidars, which are non-intrusive sensing technology.

## 1. Introduction

The aging demographic landscape presents a significant challenge for healthcare systems worldwide. As life expectancy increases, so does the prevalence of age-related health issues, particularly those associated with falls and mobility impairments. According to the World Health Organization (WHO) [1], falls are the second leading cause of unintentional injury deaths globally, accounting for over 650,000 fatalities each year. Furthermore, non-fatal falls often result in severe injuries and reduced quality of life, contributing further to the immense financial burden on healthcare systems.

With that in mind, the field of human activity recognition (HAR) and fall detection has witnessed remarkable advancements, driven primarily by the rapid development and integration of sensor technologies. These advancements have the potential to improve the healthcare landscape, assist the elderly and individuals with mobility constraints, and enhance the overall quality of their life. While cost-effective solutions, like those utilizing cameras [2] and audio data collection [3], offer practicality, they simultaneously raise potential privacy concerns. Sensors, thus, have emerged as a key tool in this endeavor, providing the means to monitor, understand, and respond to human movements and behaviors within indoor environments. These sensors are at the forefront of research and technological innovation as they address the pressing need for improved healthcare, patient safety, and assisted living for an aging global population.

For several decades now, there has been a remarkable upswing in research aimed at closely monitoring the physical activity of elderly individuals and those with mobility constraints using sensors. Numerous approaches have emerged as potential solutions to tackle this challenge while accounting for a wide variety of factors and constraints, including speed; performance; ease of deployment; scalability; and, most importantly of all, the preservation of the privacy of the individuals undergoing observation.

Sensors used for HAR can broadly be divided into two distinct categories.

1. The first category comprises sensors that need to be physically attached to the individual being monitored, either directly to their bodies or as part of their clothing. These sensors commonly leverage accelerometers and gyroscopes as well as pressure sensors to detect movements across various dimensions and subsequently identify different activities. Often integrated into wearable devices like smartwatches [4] and smart clothing items [5], these attached sensors provide invaluable data. It is important, however, to acknowledge that they can be somewhat obtrusive and may influence the comfort and independence of the elderly individuals under surveillance.
2. The second category comprises sensors that do not need to be carried by the monitored individuals or attached to their bodies/clothes. Instead, they are strategically positioned within the environment where these individuals reside, such as their room or a care facility. These sensors are designed to capture diverse types of data, including heat distribution [6,7,8,9], and fluctuations and perturbations in WiFi signal propagation [10,11,12]. They offer a less invasive method of monitoring physical activity and cause minimal disruption to the daily routines and comfort of the elderly individuals. As a result, this category of sensors has gained substantial attention among researchers, aligning with the overarching goal of preserving the autonomy and well-being of the elderly.

In essence, the pursuit of effective physical activity monitoring for elderly individuals and those with mobility challenges is a multifaceted endeavor that hinges on an equilibrium between technology, privacy, cost, and comfort. Research in this field continues to evolve, with a dedication to addressing these core considerations while striving to enhance the quality of life and overall well-being of the individuals being monitored.

In a relevant context, Lidar technology (hereafter referred to as Lidar), despite its historical association with specialized applications such as meteorology; agriculture; and, more recently, autonomous driving [13,14], has traditionally been constrained by its high cost. However, there has been a notable shift over the years, marked by a significant reduction in the cost of this technology. This reduction has facilitated the expansion of Lidar’s utility across a broader spectrum of domains. In this evolving landscape, Lidar systems have garnered increased attention, particularly in indoor environments. One of their prominent applications is the detection of dynamic objects indoors. This encompasses the identification, recognition, and tracking of moving entities, whether they are humans, pets, or robots. While the field of Detection And Tracking of Moving Objects (DATMO) is not new and has been subject to extensive research, the utilization of Lidar technology for this purpose is a relatively recent development [15]. Several techniques for object localization and tracking are being explored in this context. More importantly, several research efforts have emerged, leveraging 2D Lidar-related techniques in the medical field, to enhance, for instance, gait tracking [16] and to perform HAR [17,18,19]. These developments represent a significant leap forward in the application of this technology to enhance healthcare-related activity detection.

In contrast to prior research endeavors, the primary objective of our current study is to tackle activity detection, even in the presence of obstacles. Unlike studies such as [18,20], where a limited number of obstacles were considered, our current research delves into more realistic scenarios featuring a greater variety of furnishings, akin to the layout of a typical room. Furthermore, we depart from the convention of placing the Lidar sensor in the center of the room, where the monitored individual may be walking. Instead, we position the Lidars near the room’s periphery similar to how they would be placed in real-world scenarios, to avoid contact with the elderly and thus enhance the robustness of our approach. Finally, with the growing trend of eXplainable Artificial Intelligence (XAI), our approach is meant to introduce a representation of the data collected by the Lidar that humans find easy to grasp, giving more trustworthiness to our Artificial Intelligence (AI) model and more generally to the overall proposed approach.

The key contributions of this paper can be summarized as follows:

- We introduce a novel approach that employs multiple 2D Lidar sensors, integrating their data to perform three distinct classification tasks: activity detection, fall detection, and identifying unsteady gaits.
- We perform an early fusion of the collected data (i.e., during the processing phase), providing a series of image-like input to our neural network classifier.
- We present an innovative representation of 2D Lidar data, subject to thorough experimentation to reveal its considerable potential. The representation is nonetheless easily interpretable by humans, allowing our approach to gain trustworthiness, even among non-ML experts.
- Leveraging this representation, we train a Convolutional Long Short-Term Memory (ConvLSTM) neural network for real-time activity detection.
- We evaluate our approach under different settings and identify the optimal setting for better performance.

Through our comprehensive experimentation, we establish that the novel method and representation can surpass the performance of conventional techniques in this domain.

The structure of this paper is outlined as follows: In Section 2, we provide an overview of related works that pertain to our research. Section 3 serves as the foundational framework for our proposed method, where we establish key concepts, ideas, and naming conventions. In Section 4, we offer a comprehensive description of our proposed approach, delving into its specifics. Section 5 outlines the specifications of our simulations and offers a detailed account of our implementation. Section 6 showcases our experimental results, providing insights into the outcomes of our research. Finally, in Section 7, we conclude our paper and lay out potential directions for future work.

## 2. Related Work and Motivations

### 2.1. Related Work

#### 2.1.1. Lidar Technology

With the decrease its cost over the years, Lidar technology has become affordable for regular consumers, and its usage has expanded into various domains. Nonetheless, the mastery of this technology came with a decrease in transmission power, making it safe to use for humans and living things. Within this evolving landscape, Lidar has garnered increased attention in indoor environments. One prominent area where Lidar has shown promise is in the detection of dynamic objects within indoor spaces. This term encompasses the identification, recognition, and tracking of moving entities, whether they be humans, pets, or robots. Several localization techniques have been developed for this purpose. Some employ probabilistic methods aimed at clustering data points and determining the changing positions of these clusters over time, as seen in works like [21]. Similarly, for dynamic environment mapping, works such as [22] propose probabilistic techniques for identifying and isolating dynamic objects to represent them in 3D space. Nonetheless, one of the most widely adopted techniques is frame-to-frame scan matching, initially pioneered by Besl et al. [23] and further refined in subsequent works like [24,25]. Frame-to-frame scan matching involves determining the relative differences between two consecutive frames, with overlapping mappings, providing a means to track dynamic objects. These methods are commonly applied in the field of robotics to enable autonomous robots to navigate specific environments. However, these techniques of localization and identification have seen applications in other fields such as the medical field for tasks such as human monitoring and human activity recognition (referred to as activity detection, as well).

#### 2.1.2. Activity Detection

Monitoring the activities of elderly individuals and those with disabilities and/or limited mobility has become a prominent subject of study, attracting the attention of researchers in both academia and industry. Various technologies and methods aiming to perform this task (i.e., activity monitoring) have been introduced in the literature, taking into account factors such as cost-effectiveness, performance, ease of implementation, scalability, and privacy preservation.

However, cost-effective approaches employing technologies like cameras [2] or audio data [3] have stirred privacy apprehensions. Consequently, there has been a growing emphasis on alternative methods that are less invasive in terms of privacy. These alternatives predominantly revolve around the use of sensors. As previously stated, sensors can be broadly classified into two categories: attached sensors, which are integrated into devices worn by individuals (e.g., those with accelerometers and gyroscopes as seen in the work of Zhuang et al. [4]), and non-attached sensors, which capture data from the surrounding environment (e.g., monitoring heat distribution and Wi-Fi signals such as in [6,7,8,9,10,11,12]).

In the former category, approaches such as that of Ha and Choi [26] and Mishkhal [27] were proposed, in which the recognition of the activity relies on accelerometers attached to the body. Approaches such as that of Webber and Rojas [28] proposed using smartphones attached to different parts of the body instead. Similarly, Barna et al. [29] included other sensed data such as humidity and temperature, in addition to gyroscope data, to recognize the human activities.

However, the preference for approaches centered on non-attached sensors stems from their ability to reduce the burden on individuals being monitored and to address privacy concerns more effectively. While each sensor type has its own set of advantages and limitations, the paramount considerations revolve around privacy preservation and user-friendliness. As a result, the research community has gravitated towards non-attached sensors as the focus of their endeavors.

Infrared (IR) array sensors, for instance, are sensors that can capture the heat emitted by the human body and map it into a low-resolution image-like format for processing. Approaches for human recognition and activity detection using IR array sensors include those of Yang et al. [6], Burns et al. [30], and Bouazizi et al. [9,31]. While IR infrared sensors achieve impressive results (i.e., over a 92% F1-score in the case of [6]) while not compromising on aspects such as privacy and cost, they have a few limitations of their own. On the one hand, they are very sensitive to noise and the presence of any heat-emitting sources such as electronic devices (computers, stoves, etc.). On the other hand, the presence of obstacles limits their potential as they block the IR rays from reaching the sensors. Finally, for good detection, these sensors need to be placed strategically to minimize their deployment cost as they have very limited coverage, and multiple sensors need to be placed in a single room to guarantee full coverage.

Frequency-modulated continuous-wave (FMCW) radars have attracted similar attention given their ability to identify activities, in particular falling activities, while allowing one to extract vital signals such heartbeat and respiration rates. Shahzad et al. [32] used such radars to recognize 7 different activities in an unconstrained environment, with an accuracy reaching 91%. Saeed et al. [33] proposed a similar approach in which they extracted spectrograms of short recordings of FMCW radars (i.e., between 5 and 10 s), which they processed via a Residual Neural Network (ResNet) to classify 6 activities with 85% accuracy in unseen environments and 96% in seen ones. Shah et al. [34] used 2 machine learning algorithms including Support Vector Machine (SVM), k-nearest neighbour (kNN) for classification, and AlexNet [35] for feature extraction. They reached 81% accuracy in activity recognition for 4 different scenarios.

Lidar technology, the focus of this work, has also been explored in the literature as a candidate for a non-intrusive robust method for activity detection-related tasks such as fall detection. Several studies have explored the application of 2D Lidar techniques for purposes such as gait tracking and human activity recognition [16,17,18,19].

Building upon the research conducted by Piezzo et al. [36] and Li et al. [37], Duong et al., in their work [16], employed 2D Lidar technology for tracking the gait of individuals who use walkers as part of gait rehabilitation. More recently, researchers like Luo et al. [17] and Bouazizi et al. [18,19] have harnessed Deep Learning (DL) algorithms to achieve activity detection using 2D Lidar technology.

In addition to 2D Lidars, 3D Lidars have also been recently explored more intensively. Works such that of Roche et al. [38] and Benedek et al. [39] have shown significant results in HAR and Gait analysis tasks, reaching an accuracy over 90% for the first and over 80% for the second.

### 2.2. Motivations

In contrast to prior research, our current study addresses 3 main challenges:

- The challenge of detecting activities even in the presence of obstacles: Unlike the study by Bouazizi et al. [18], which utilized a limited number of obstacles, our present work delves into more realistic scenarios, simulating environments with additional furniture, akin to a typical room layout.
- The challenge of real-world applicability: Unlike previous works [17,18,20], we assume that the placement of the Lidar should be near the room’s periphery rather than in the center, where individuals are more likely to walk.
- The challenge of model interpretability: With the rapid growth of AI technologies, similarly growing concern regarding the trustworthiness of AI models has surfaced. Explainable AI (XAI) has emerged as a way to build trust towards AI models by rendering them understandable to non-experts. In this context, rather than inputting an ambiguous input to our AI model and performing different sorts of fusion, rendering the model a black box, we aim to simplify the input so that the human user can tell what to expect and how the model makes the decision.

In our earlier work [40], we conducted preliminary experiments to investigate the potential of our proposed methods in detecting the activities in indoor environments and to what extent they can recognize falls from other activities. In the current work, we conduct deeper experiments and an analysis of the proposed method. We evaluate it against existing methods and highlight its superiority. We run our experiments in more diverse scenarios and under different settings. We conduct a set of experiments to identify the optimal set of settings and parameters that yield the highest accuracy. We evaluate the robustness of our proposed method against the presence of obstacles such as furniture to better estimate its utility in real-world applications.

Our results indicate that this novel approach and representation have the capability to surpass traditional methods in terms of performance and robustness.

## 3. Overall System Description

### 3.1. Equipment Used

A Lidar is a device utilized to gauge the distance to objects by emitting an infrared (IR) light beam and precisely measuring the time it takes for this beam to rebound. While different types of Lidar exist, in our particular study, we focus on a 2D Lidar. This specialized device is equipped with a unidirectional Lidar unit perched atop a motor that is continuously rotating, allowing the emission and reception of light beams in 360°. It is this type of Lidar that we rely on for our research, where we tackle three distinct yet interrelated tasks: activity detection, fall detection, and unsteady gait detection.

Within our research framework, multiple Lidar sensors are deployed continuously, collecting a multitude of measurements. These individual data streams are fused at the processing stage and concatenated to construct a comprehensive representation of the environment and its dynamic transformations. This process essentially creates rough but effective snapshots of the surroundings and their evolving states.

It is this composite view that enables our AI model to identify, isolate, and analyze changes associated with human activities. By scrutinizing these changes, our model learns to identify valuable information that it employs and processes for the sake of performing the three tasks we have set out to accomplish.

### 3.2. Naming Convention

In the current paper, we use a terminology similar to that of [18,19,20]. However, a few alterations were made to adjust to our simulation settings. These alterations are as follows:

#### 3.2.1. Measurement Point

A measurement point *p* is one distance measured for an angle θ at a given time *t* by a Lidar $L^{l}$ . Therefore, each measurement point is indexed by the identifier of the Lidar that made it. As such, the measurement point could be expressed as $p^{l}\left(t\right)=\left(r\left(t\right),\theta \left(t\right)\right)$ , where r(t) is the measured distance and θ(t) is the angle of measurement at the instant *t*. For simplicity, when referring to a measurement point, we drop the index *l* and keep the index when referring to scans made by the Lidar *l*, as we will describe below.

#### 3.2.2. Scan

A scan is the set of measurement points collected by a Lidar during a single rotation. Since speed of rotation of Lidars is typically very high (i.e., 10∼100 Hz), it is fair to assume that all measurement points of a single scan are taken at the same point in time *t*. With that in mind, a scan $S^{l}\left(t\right)$ could be expressed by

$$S^{l}\left(t\right)=\left\{p_{i}\left(t\right)=\left(r_{i}\left(t\right),\theta_{i}\left(t\right)\right),\;\;\;\;\;\;i=\left[1,\cdots ,N\right]\right\},$$

where $p_{i}$ is the ith measurement point, and *N* is the normalized number of measurement points collected during a single rotation of the Lidar (i.e., scan). While for technical reasons, Lidars collect different number of measurement points in each rotation, we normalize the scans via interpolation to have exactly N=360 so that for each 1°, a measurement point is recorded (i.e., $\theta_{i}\in \left\{0^{\circ },1^{\circ },\cdots ,359^{\circ }\right\}$ ).

#### 3.2.3. Full Scan

A full scan F(t) refers to the concatenation of different scans collected by all the Lidars at the same instant *t*. In other words, given a set of $N_{L}$ Lidars placed in an area of interest (e.g., a room), the full scan of this set of Lidars at an instant *t* can be represented as follows:

(1) $$F\left(t\right)=\left\{\begin{array}{c} S^{1}\left(t\right)=\left\{p_{i}\left(t\right)=\left(r_{i}\left(t\right),\theta_{i}\left(t\right)\right),\;\;\;i=\left[1,\cdots ,N\right]\right\},\\ S^{2}\left(t\right)=\left\{p_{i}\left(t\right)=\left(r_{i}\left(t\right),\theta_{i}\left(t\right)\right),\;\;\;i=\left[1,\cdots ,N\right]\right\},\\ \vdots \\ S^{N_{L}}\left(t\right)=\left\{p_{i}\left(t\right)=\left(r_{i}\left(t\right),\theta_{i}\left(t\right)\right),\;\;\;i=\left[1,\cdots ,N\right]\right\}, \end{array}\right.\;$$

#### 3.2.4. Map

As previously stated, once data (i.e., scans) are collected by all the Lidars, the data are concatenated all together and converted to a more understandable data format (i.e., 2D grid) prior to classification. At a given time *t*, the process of conversion of a set of full scans F(t) from $N_{L}$ Lidars, which are taken at the same time *t*, into a 2D grid is referred to as $T\left(F(t)\right)$ , and the generated map is referred to as M(t) . In other words,

(2) $$M\left(t\right)=T\left(F(t)\right).$$

Later on, we will elaborate on our process for converting the set of scans into a 2D grid. It is essential to point out that when $N_{L}$ equals 1, a single Lidar is being used, resulting in a map comparable to what a single Lidar can produce. This partially resembles the case scenarios of out previous work [18]. However, our transformation method made in the current work shows a significant improvement in performance compared to the previous one in realistic scenarios. This distinction is crucial because we will be comparing the outcomes achieved with multiple Lidars against those achieved with a solitary Lidar using both our newly proposed method and our previous one introduced in [18].

### 3.3. System Description

The methodology presented in [18] hinges on a single Lidar for the dual tasks of pinpointing an individual’s location and monitoring their activities. However, this approach necessitates a careful placement strategy to minimize areas where the person might become obscured by obstructions. Moreover, the precision of locating the person is compromised due to the specific data collection method employed by the Lidar, as elaborated upon in [18]. More specifically, Lidars can collect data up to the closest point of contact with the object being monitored: When a person’s data are collected, only their side facing the Lidar is detected and identified. The center of the human body (or any other object for that matter) is not identifiable, and the location is usually either set to the centroid of the detected points or estimated using some interpolations. To surmount these inherent limitations, our current research leverages the use of multiple Lidars, positioned at various locations, to collaboratively identify both the person’s location and their ongoing activities within a given room more accurately.

Within our study, we employ simulated environments featuring diverse room layouts, complete with furniture, and individuals engaged in a wide range of activities, encompassing normal and atypical walking, sitting, falling, lying down, and more. A visualization of a simple scenario is given in Videos S1 and S2 in the Supplementary Materials.

**Figure 1:**
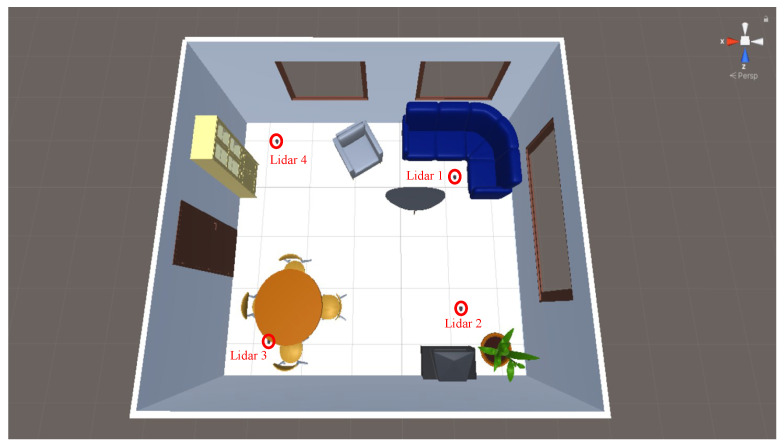
A caption of one of the environments in which the simulations are run. In red, the positions of Lidars used are marked.

The Lidars we employ are arbitrarily indexed, and the Lidar given “index 1” is the one positioned to the north, while the subsequent Lidars are indexed clockwise in ascending order. For visual clarity, we offer an illustrative example of a room layout with the placement of Lidar sensors in Figure 1.

**Figure 2:**
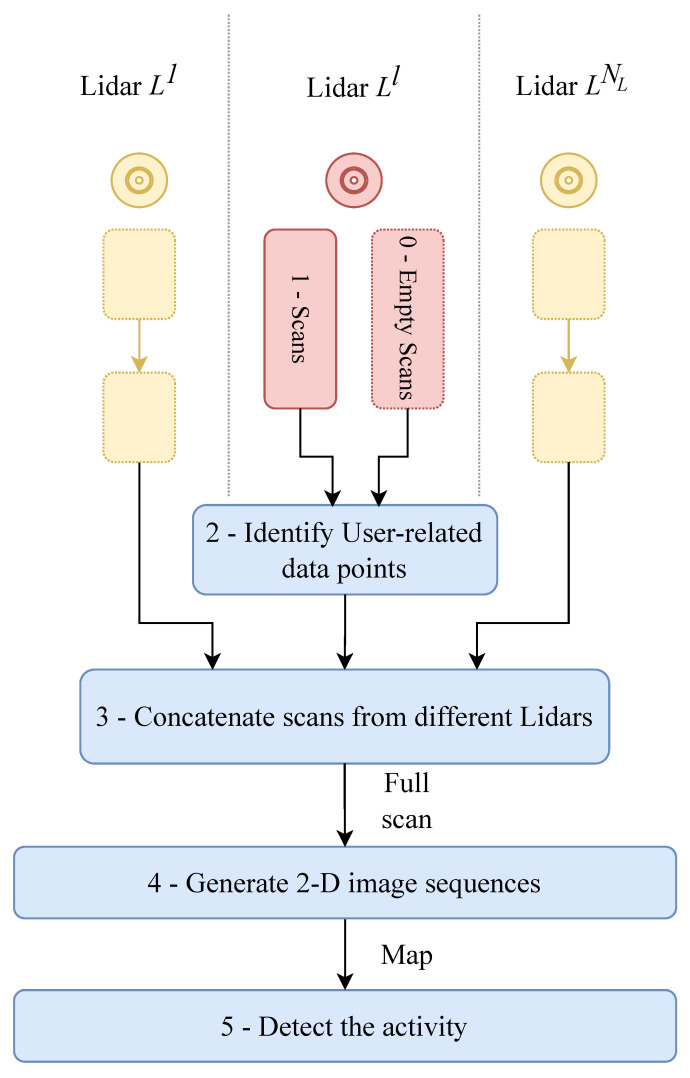
A flowchart of the proposed approach for activity detection.

Taking this into account, in contrast to the approach in [18], we do not treat Lidar data points as a time series of one-dimensional vectors, or scans. We undertake data transformation, converting them into sequences of 2D images, which we classify as such. Figure 2 illustrates our proposed approach in a flowchart.

For each room, we assume that collecting Lidar measurements is feasible when no subject is present in the room. Measurements collected in the absence of subjects are called “empty scans”. From these empty scans, gathered from various Lidars, it is possible to create what we refer to as an “empty map”. Once the empty scans and map are constructed, they serve as a reference for subsequent steps.

We carry out multiple scenarios in diverse room environments, which we will elucidate later in detail. Each scenario mimics a series of continuous, uninterrupted activities, much like how an individual would conduct their everyday tasks. For each Lidar dataset (individually), we employ a clustering algorithm with reference to the empty scans and map. The data collected from all Lidars are then combined and interpolated to provide a more robust representation. This process generates a 2D image for each time step *t*. The set of 2D images over a certain period of time is then subjected to classification via a deep learning model. Depending on the specific task being performed, an appropriate pre-trained model is invoked for the classification. These steps are comprehensively explained in the following Section 4.

## 4. Proposed Method: Detailed Description

### 4.1. Identification of User Data Points

The initial step in our proposed approach involves identifying user-related data points. This process is conducted individually for each Lidar, denoted as $L^{\left(l\right)}$ , and it draws inspiration from the technique outlined in our previous work [18]. To perform user data point identification, we refer to an empty scan in addition to the actual scans that we intend to classify. When a person enters a room and carries out their designated activities, any deviations in data points from those observed in the empty scan are typically attributed to the presence of a person. Specifically, for a given angle θ , we denote the distance recorded in the empty scan as $r_{0}\left(\theta \right)$ and the distance recorded in the real scan as r(θ) . If the absolute difference $|r\left(\theta \right)-r_{0}\left(\theta \right)|$ exceeds or equals a predefined threshold, denoted as $\delta_{Th}$ , the newly detected point is considered to be potentially indicative of the subject’s presence. To account for potential noise, we apply a modified version of the algorithm previously introduced in [18]. This modified algorithm consists of four primary steps:

1. Candidate Point Identification: Given a scan S(t) captured at a specific time instant *t* and an empty scan $S_{0}$ , all points at various angles θ that meet the condition $d\left(\theta \right)-d_{0}\left(\theta \right)\geq \delta_{Th}$ are regarded as candidate useful points. These points are then aggregated into a set denoted as $S^{*}\left(t\right)$ .
2. Clustering Using DBSCAN: We apply the Density-Based Spatial Clustering of Applications with the Noise (DBSCAN) algorithm [41] to the set $S^{*}\left(t\right)$ . This step allows us to identify clusters of data points that are likely attributable to the subject’s presence.
3. Cluster Merging and the Subject’s Location: Subsequently, the clusters found in the previous step are merged, and the centroid of the merged cluster is considered to represent the location of the subject.
4. Refinement: Once the centroid of the location of the subject is identified, refinement is carried out by selecting the data points within a certain range $\Delta_{R}$ , which are considered as being related to the subjects and are kept, whereas others are discarded.

In essence, this process allows us to identify user-related data points by comparing real scans to empty scans, effectively pinpointing areas where a person’s presence is likely, even in the presence of noise and variations.

### 4.2. Transformation of the Data

After extracting the data from each individual Lidar, we perform a transformation of their coordinates to obtain their absolute Cartesian values. In simpler terms, these points are no longer expressed in relation to the specific Lidar that captured them. To illustrate, considering a Lidar labeled as $L^{l}$ located at coordinates $\left(x^{l},y^{l}\right)$ , and a measurement point $p_{j}\left(t\right)=\left(r_{j}\left(t\right),\theta_{j}\left(t\right)\right)$ collected by this Lidar $L^{l}$ , the Cartesian coordinates $\left(x_{j},y_{j}\right)$ of the point $p_{j}\left(t\right)$ can be expressed as follows:

(3) $$\begin{array}{ccc} x_{j}\left(t\right) & = & x^{l}+r_{j}\left(t\right)\cos\left(\theta_{j}\left(t\right)\right),\\ y_{j}\left(t\right) & = & y^{l}+r_{j}\left(t\right)\sin\left(\theta_{j}\left(t\right)\right). \end{array}$$

**Figure 3:**
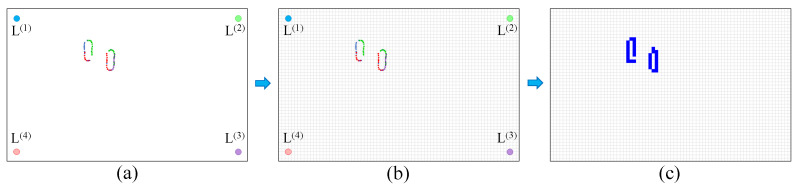
The steps for the creation of the 2D images from the concatenation of the different Lidar data. (**a**) shows the concatenation of the data points, (**b**) shows the placement of the grid, and (**c**) shows the process of fitting the data points to the grid.

Now detached from their original Lidar sources, the lists of user-related data points are combined and collectively utilized to generate 2D images. In Figure 3, we provide a concise overview of the process for creating 2D images from the Lidar scans. In this procedure, after data points have been clustered, and only those data points associated with the subject have been singled out (as illustrated in Figure 3a), the coordinates of these detected points are converted into the absolute Cartesian coordinate system, taking into account the known locations of their respective Lidars. Once all the points are converted into this new coordinate system, a 2D grid is superimposed onto the room, as depicted in Figure 3b. Pixels within the grid cells where at least one data point is located are assigned a value of 1 (represented in blue in Figure 3c), while the rest are assigned a value of 0. This process results in the creation of a 2D image for each time step. By monitoring changes in the identified data points over time, our objective is to recognize the specific activity being performed.

In our simulation experiments, we assessed this image generation technique using various grid sizes. Larger grids produce larger and more detailed images, while smaller grids generate smaller, less detailed images. In our simulations, we employed three grid sizes: 60 × 40, 90 × 60, and 120 × 80, yielding sequences of images with the same dimensions.

### 4.3. Activity Detection

As previously discussed in Section 1, the process of activity detection entails categorizing the actions performed by subjects, irrespective of their location. In order to maintain consistency and facilitate equitable comparisons with established methods, such as those presented in [17,18], we undertake the following sub-tasks:

- (T1) Activity Detection: This sub-task aims to determine the specific activity being carried out by the individual. It encompasses the recognition of five distinct activities, which include walking, standing, sitting on the ground, falling, and lying down.
- (T2) Fall Detection: Given that falling is the only potentially hazardous activity, the primary objective is to distinguish instances of falling from all other activities. This task involves binary classification, with all activities, excluding “falling”, grouped into a single class.
- (T3) Unsteady Gait Detection: Identifying an unsteady gait is essential as it may indicate potential hazards such as a heart attack or an increased risk of falling. This task assesses the capability to detect a steady gait versus an unsteady gait.

These sub-tasks collectively constitute the main sub-tasks for our activity detection methodology and form the basis for our evaluation and comparison with existing approaches.

**Figure 4:**
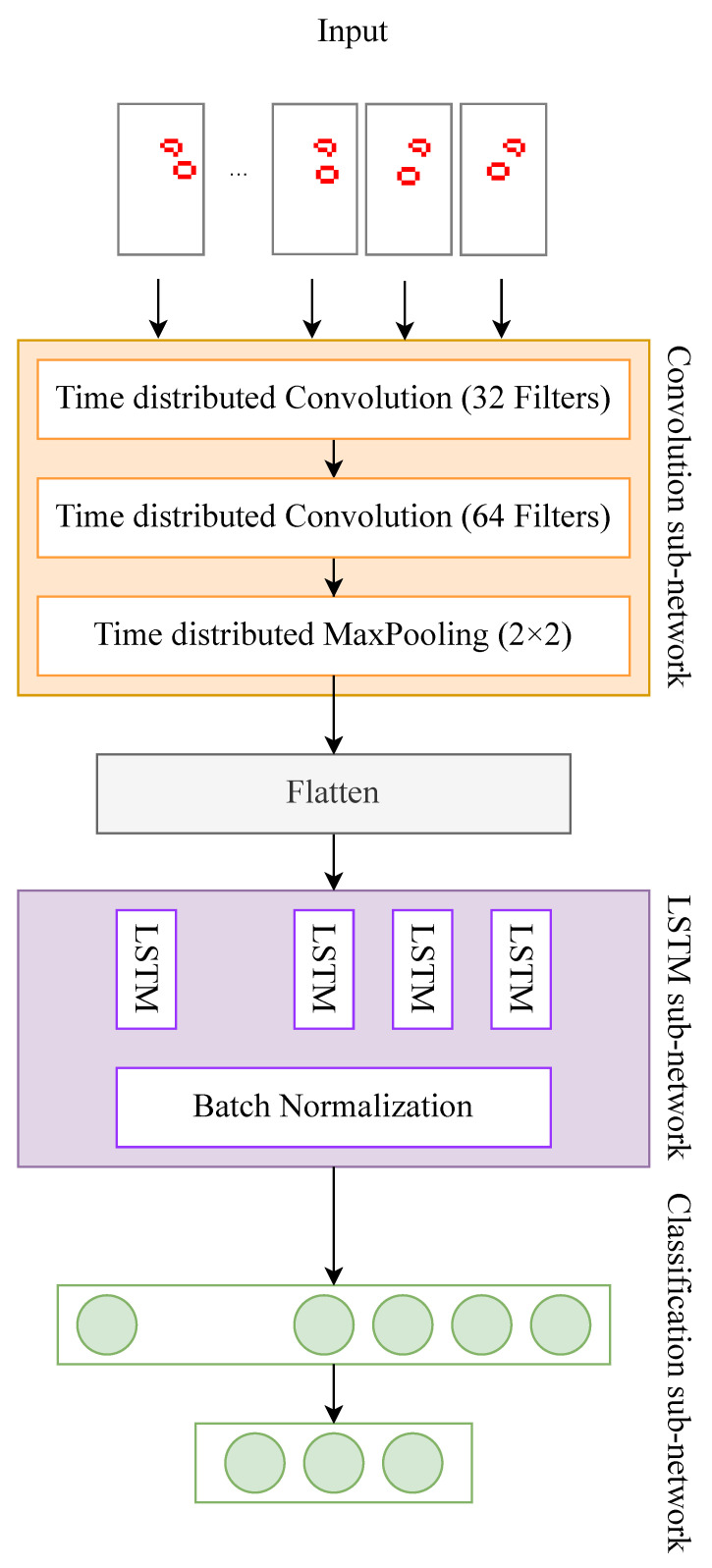
Architecture of the ConvLSTM neural network used for classification.

To address the sub-tasks, a consistent approach is applied. These sub-tasks are formulated as classification problems and are resolved through the utilization of deep learning methodologies. Specifically, the input for the classifier is structured as a sequence of images, ideally suited for a Convolutional Long Short-Term Memory (ConvLSTM) neural network architecture. The architectural design employed in our study is illustrated in Figure 4 and consists of the following components:

1. Two time-distributed convolutional layers, with 32 and 64 filters of dimensions 3 × 3, sequentially followed by a 2D 2 × 2 MaxPooling layer.
2. Subsequently, the data are flattened and fed to an LSTM layer equipped with 200 units.
3. The output of the LSTM layer undergoes batch normalization and is fed to two consecutive dense layers: the first layer encompasses 128 neurons, while the second layer has $N_{C}$ neurons, with $N_{C}$ denoting the number of classes.

As previously mentioned, the classification task is executed either as a five-class classification (activity detection) or as a binary classification (fall detection or unsteady gait detection). The convolutional component of the network is responsible for extracting features from the input images, independent of the temporal interrelationship between them. In contrast, the LSTM segment of the network captures temporal variations in these features over time.

It is important to note that despite the varying sizes of the input images, the convolution layers share the same number and size of kernels. The primary distinction is in the size of the input layer, which is a technical detail, and the dimension of the input provided to the LSTM layer, which is calculated automatically.

## 5. Simulation Specifications and Implementation

### 5.1. Simulation Specifications

Our simulator was developed using Unity, a versatile game engine introduced by Haas in 2014 [42]. Unity serves as a platform that empowers creators to design 2D and 3D games; design coding functionalities in C#; and integrate a wide variety of assets such as textures, 3D models, and lighting objects, often generated using various external tools. Over the years, Unity’s applications have extended beyond gaming as it supports multiple physics engines capable of replicating real-world object behaviors, focusing on three crucial aspects: determinism, repeatability, and performance. For example, Unity has been adopted in diverse fields beyond gaming, including the development of simulators for robotics and for autonomous vehicles, as detailed in works like [43,44]. In our simulator, we leverage the raycast and ray tracing features of PhysX, the foundational physics library within Unity, to replicate the work and operation of 2D Lidars.

The comprehensive details of our simulator’s construction are introduced and explained in a separate work [20]. However, in this work, we provide an introductory overview of the implementation. We procured a wide range of 3D models and animations depicting various activities from multiple sources, facilitated by the efforts of Naureen et al. [45]. Their extensive database includes over 11,000 animations spanning more than 300 subjects, categorized into classes, facilitating the selection of relevant animations for our purposes. Additionally, we utilized SweetHome3D to create 3D indoor environments complete with various types of furniture. By importing these created assets into Unity, we were able to generate 3D animations simulating realistic human movements within indoor settings. To simulate the behavior of the 2D Lidar, which casts rays in different directions while rotating, we integrated the Robot Operating System (ROS). The Unity ROS Simulator enabled communication between Unity and ROS, facilitating the collection and transmission of data and measurements obtained by the 2D Lidar.

It is noteworthy that real-world 2D Lidars exhibit a range of limitations, including inaccuracies and variations in time steps between consecutive measurements. Therefore, in our simulations, we intentionally introduce some of these realistic patterns, adhering to the simulation specifications detailed in Table 1.

### 5.2. Experiment Design

Throughout our simulations, we aim to show the performance of our proposed method, as well as which parameters are best for our method to work. Therefore, the data collected are meant to address four main goals:

1. Identify the optimal fusion strategies,
2. Identify the optimal grid size,
3. Identify the optimal Lidar height,
4. Show the performance of our proposed method compared to existing ones,
5. Highlight the obstacle resilience of our approach.

Our experiments are, therefore, designed as follows.

#### 5.2.1. Fusion Strategies

The primary goal of this experiment is to compare different fusion strategies for combining data from multiple Lidar sensors to demonstrate the usefulness of early fusion of date over late fusion, voting, or the use of a single Lidar sensor. The choice of fusion strategy can significantly impact the accuracy and reliability of human activity recognition and unsteady gait/fall detection systems. Early fusion combines raw data from multiple Lidars into a single representation before processing, late fusion processes the data separately and then fuses the results, and voting aggregates decisions from multiple Lidars. These strategies have different implications for performance and computational efficiency.

#### 5.2.2. Optimal Grid Size

This experiment aims to determine the best grid size that maximizes the detection accuracy. As previously stated, larger grids produce larger and more detailed images, while smaller grids generate smaller, less detailed images. However, larger sizes make the detected points sparser, leading the convolution part of our neural network to miss important features related to the adjacency of pixels. Thus, it is important to identify the optimal grid size that has a balance between the level of details and the proper functioning of the neural network.

#### 5.2.3. Optimal Lidar Height

This experiment aims to determine the optimal height placement of Lidar sensors to maximize the accuracy of activity recognition and fall detection. The height of Lidar sensors can influence their field of view, coverage, and ability to capture relevant information. Finding the ideal height is crucial for achieving high detection rates.

#### 5.2.4. Comparative Study

Once the fusion strategies, the grid sizes, and the optimal Lidar heights are compared, the goal of our next set of experiments is to showcase the superiority of the proposed method for activity recognition and fall detection using multiple Lidar sensors when compared to existing methods in the field. Demonstrating the superiority of the proposed method is critical for establishing its relevance and competitiveness in the field of human activity recognition and fall detection.

#### 5.2.5. Obstacle Resilience

The goal of this experiment is to assess the resilience of the proposed method to the presence of obstacles within the environment and demonstrate that it can effectively handle situations where obstacles partially obstruct the view of Lidar sensors. This is because real-world environments are often cluttered with obstacles, and it is essential to ensure that the method remains effective even when Lidar sensors’ lines of sight are partially blocked.

### 5.3. Data Sets

Despite the different sets of experiments and their goals as indicated in the previous subsection, the data sets created are for the most part made with the same simulation conditions and in the same environments. For instance, when conducting the activity detection for different heights of the Lidar, the same activities as collected from the AMASS data set [45] are used, and the same simulated rooms are used. These same activities and environments are also used to compare our method to the existing ones.

In total, our simulations were run in nine different indoor room environments. Each room has its own furniture placed in typical positions (e.g., sofas and closets are placed near the walls, tables are closer to the center, etc.). Nine different human models were also used, whose heights, body shape, and gender were not selected with specific ranges but randomly picked from the library. Five male and four female models were selected. The human models as well as the rooms used for training and testing our method were exclusive to their respective data set. In other words, a given room and a given subject’s collected data are used either for training or for testing. This allows for a better evaluation of our model in unseen environments and unseen subjects. It is important to note, however, that many of the furniture 3D models are reused in different rooms, though placed differently. The subjects were continuously carrying out all sorts of activities for a few minutes. Some of the activities, mainly the “walk” and “sit down” activities, despite being reused by subjects, differ from one subject to another in two ways: the speed at which they are performed is different, and the angles with reference to the Lidar’s positions are different. In addition, given the different heights of the subjects, this results in different step sizes and leg spread. In other words, even though these activity assets are reused, we believe they have no big influence on the results of the classification. The recorded data are then cropped to the same length, eliminating transitions between the activities to create the different samples.

With that in mind, Table 2, Table 3 and Table 4 show the structure of the data sets generated using the simulator. In particular, the data set generated for the task (T2) is a sub-set of that used in (T1). Roughly, for each activity, 500 samples were generated as part of the training set, and 400 were generated as part of the test set. This resulted in a total of 2500 training and 2000 test samples for the data set used for (T1), and 1000 training and 800 test samples for the data set used for (T2) and that used for (T3).

The data sets are formatted as previously described to contain the following information for each scan *S* at a given instant *t*:

- The scenario to which the scan belongs,
- The time *t* of each scan,
- The position of the four Lidars,
- The full scans collected by the different Lidars, indexed by their respective indexes and containing the polar coordinates with reference to their respective Lidar’s position.

## 6. Simulation Results

### 6.1. Simulation Visualization

**Figure 5:**
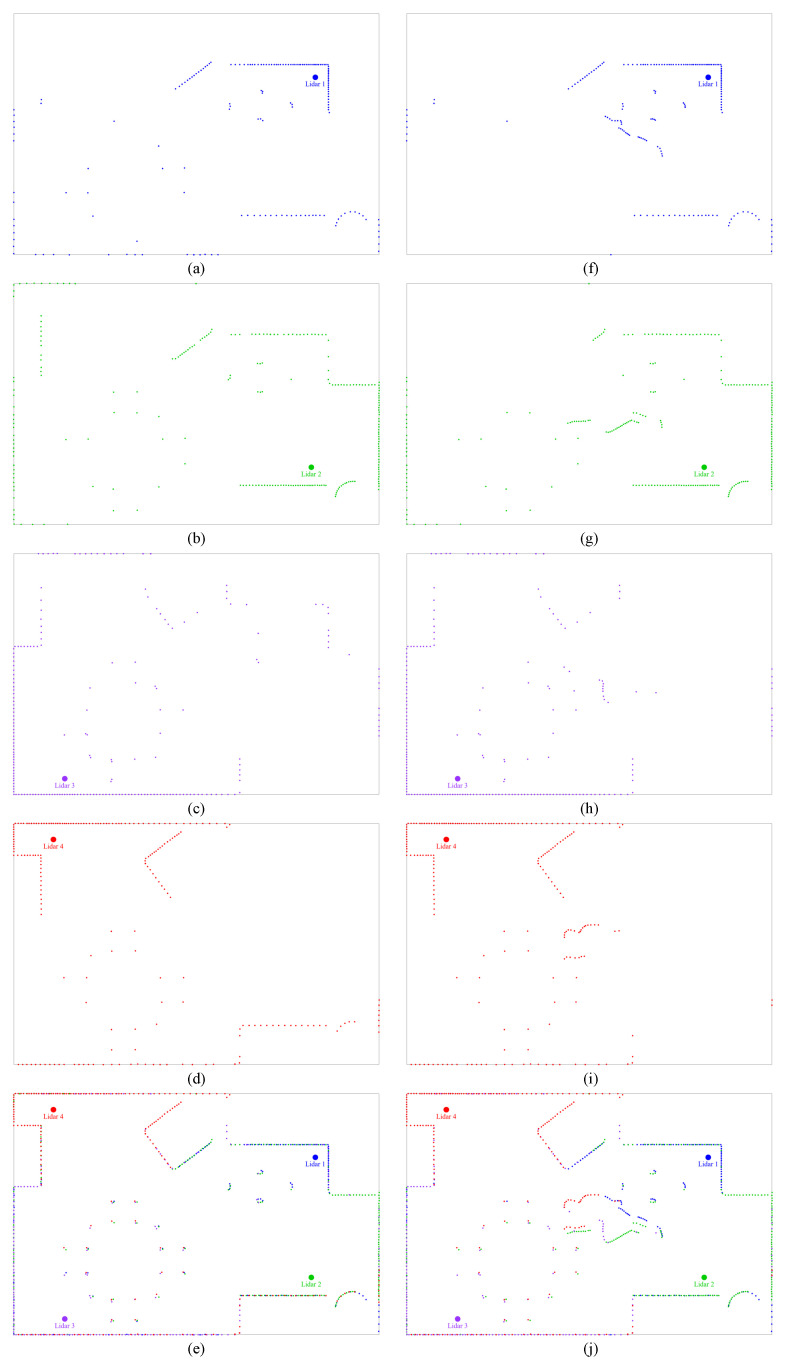
Visualization of the data collection and concatenation in presence and absence of the subject. Panels (**a**)–(**d**) show the data collected by the individual Lidars when the subject is absent; (**e**) shows their concatenation; Panels (**f**)–(**i**) show the data collected by the individual Lidars when the subject is present; and (**j**) shows their concatenation.

In this subsection, we visualize the output of the simulations, namely, the Lidars’ generated data, the output of the fusion process, and the output of the person points identification. In Figure 5, we show the data collected by the four Lidars individually in the absence of the subject—sub-figures (a), (b), (c), and (d). In sub-figure (e), we show their concatenation, which gives a decent visualization of the shape of the room previously shown in Figure 1. This map obtained after concatenation serves, as we referred to previously, as the “empty map”. By comparing this map to the one generated in the presence of a subject, we can easily identify the points generated due to this presence. This can be clearly seen in the sub-figures (f), (g), (h), (i), and (j). The former four sub-figures show the data collected by the individual Lidars, whereas the latter one shows the concatenation of their output.

**Figure 6:**
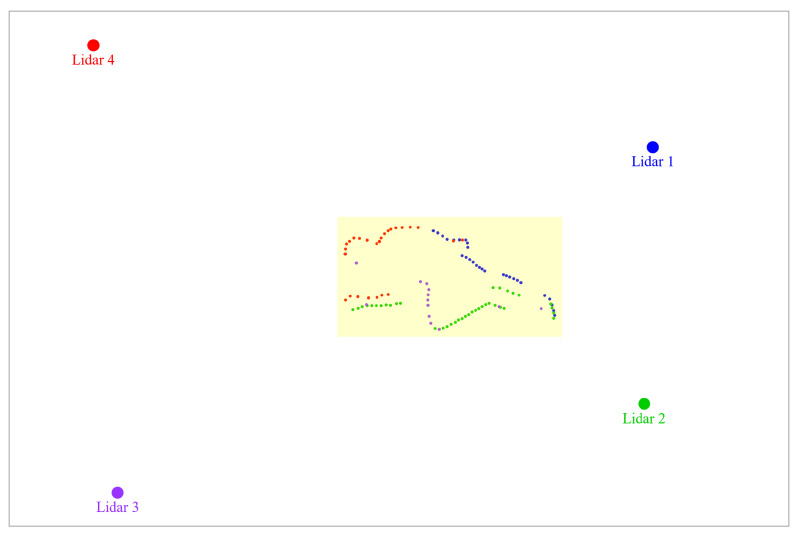
Visualization of the results of the process of isolation of data points generated due to the presence the subject.

In the Figure 5j, we can see the new data points collected, forming the shape of a person lying on the ground. This is indeed the case for this particular frame. In Figure 6, we use the clustering process to isolate the data points created due to the subject’s presence. The subject’s overall body is highlighted in yellow (the highlighting serves only for visualization and is not part of the approach).

**Figure 7:**
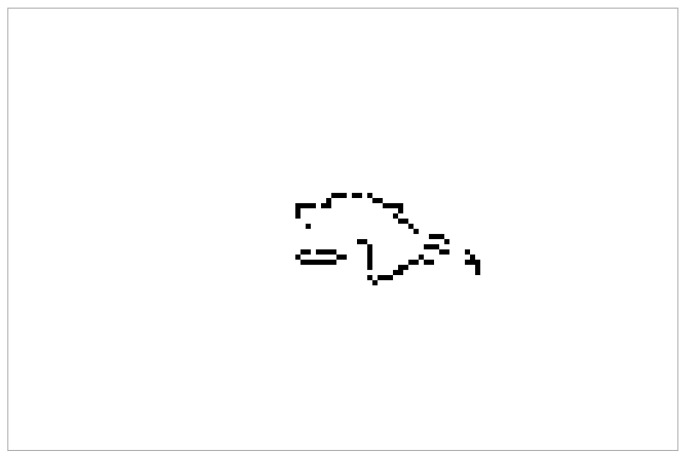
Visualization of the image generated after transformation of the data points into an image.

By applying the conversion technique that we described in Section 4.2, we obtain the image shown in Figure 7. Similar images are generated at every frame and serve as input to our LSTM network for classification. 

Throughout the rest of this section, we will be using four key performance indicators (KPIs): accuracy, precision, recall, and F1 score. These metrics collectively provide a comprehensive assessment of our method’s performance and effectiveness.

### 6.2. Settings Optimization

As previously stated, our first goal is to identify the settings for which the classification accuracy is the best. With that in mind, we run our first round of experiments solely on the task (T1) data set and report the results for this particular tasks. A few reasons lay behind this choice. First of all, the network trained for (T1) is later used for transfer learning to fine-tune it (except for the last layer) on the other two tasks. In addition, this task exhibits a higher number of classes. Therefore, good classification performance for this task guarantees similar performance for the other two, whereas the opposite is not necessarily true. Finally, distinguishing between normal gait and unsteady gait, for instance, does not reveal the performance of the identification of height-sensitive classes, such as when a person is laying on the ground.

In the rest of this subsection, we aim to identify the optimal settings, which we use in the subsequent subsections.

#### 6.2.1. Fusion Strategy

The goal of this set of experiments is to compare our method of early data fusion with an interpretable representation of late fusion or voting systems, where the data collected from the different Lidars are processed through different neural network branches.

**Figure 8:**
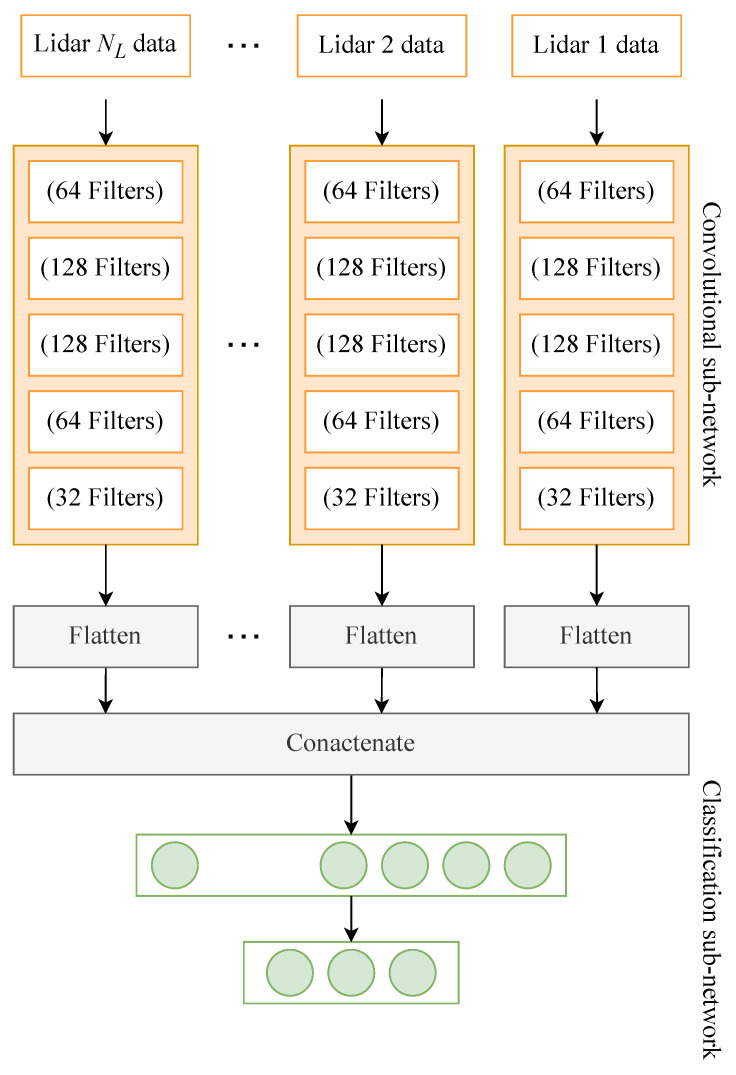
Architecture of the neural network used for classification in the late fusion approach.

For this sake, we evaluate our neural network architecture against the one shown in Figure 8 for the task (T1). In this architecture, after cleaning and identifying the subject-related data points, each Lidar datum is transformed into 2D images and input separately to a convolutional sub-network composed of five convolutional layers with [64, 128, 128, 64, and 32] filters, respectively. These data undergo similar processing before they are concatenated and passed through three dense layers with 100, 50, and $N_{C}$ neurons. Note that the input images to this network are not ones made from the concatenation of the data but rather are created in a similar way to [18], were the data from different angles are flatteneted and stacked for every time step to create a single image.

For consistency with what comes next, the Lidar height and the grid size are set to their optimal values, i.e., 35 mm from the ground, and 90 × 60, respectively.

The results of classification for the early data fusion against those for the late fusion are given in Table 5. As can be seen in the table, carrying out the early fusion and transforming the data the way we did yielded much higher accuracy than when processing the Lidars’ data separately before concatenating them for classification.

#### 6.2.2. Optimal Lidar Height

In this subsection, similar to our previous experiments, we evaluate the classification results when placing the Lidars at different heights. For this sake, again, we use the optimal grid size (i.e., 90 × 60); employ our early fusion technique; and adjust the height of the Lidar each time to be at one of the following values: 5 mm, 35 mm, 100 mm, 200 mm, and 250 mm.

We use the same technique proposed in Section 4 and use the same neural network introduced in Figure 4. For the different used height values, we obtained the overall accuracy, precision, recall, and F1 score shown in Table 6.

**Figure 9:**
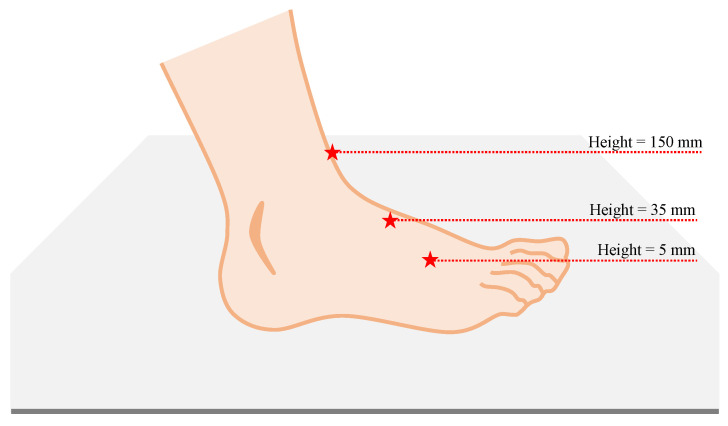
A visualization of how the point hit by the Lidar ray may differ at different heights.

As can be seen, for the lowest values of height (i.e., 5 mm and 35 mm), the results are very close to one another, with the accuracy reaching 96.32% for a height equal to 5 mm and reaching 96.10% for a height of 35 mm. With the height increases, we notice a clear drop in the performance of the classification. This is particularly due to the fact that with such an increase in height, a large portion of the information gets lost. In Figure 9, we show a visualization of how the point hit by the Lidar ray may differ at different heights. At lower heights, the shape of the feet can be detected, unlike for higher heights. Nonetheless, when a person is walking and lifting their feet from the ground, such an action can be detected when the Lidar is placed at a lower height, which cannot be said for the higher heights. This renders the process of detection of an unsteady gait, in particular, hard. More importantly, when the subject falls and lays on the ground, for better detection of the shape of a person laying on the ground, the Lidars need to be placed at lower height. This explaines why for the lowest values of height (i.e., 5 mm and 35 mm), the results are better and are very close to one another.

That being said, realistically, Lidar systems used in real-life do not offer heights equal to 5 mm. Nonetheless, their being very close to the ground makes them very susceptible to noise and to reflections from nearby objects on the ground. In the literature, the RPLidar A3M1, a 2D Lidar manufactured by Slamtec (https://www.slamtec.ai/product/slamtec-rplidar-a3/ (accessed on 18 November 2023)), has been commonly used. When placed on the ground, the height of the emitter and receiver is 35 mm, which was the reason we evaluated our system for this particular height. Therefore, we believe that the performance obtained for this height is more realistic and useful for real-world applications. As a result, hereafter we will be using the height 35 mm, despite not offering the highest accuracy.

#### 6.2.3. Optimal Grid Size

In this subsection, we undertake a comparison of classification results for the task (T1) while employing various grid sizes. To recap, we utilize three distinct grid sizes: 60 × 40, 90 × 60, and 120 × 80. Table 7 presents the overall accuracy, precision, recall, and F1 score of the proposed method for each of these grid sizes. As can be seen, the highest accuracy is obtained when employing a grid with a size equal to 90 × 60, reaching 96.10%. It becomes evident that employing higher 2D image resolutions does not necessarily enhance the accuracy of detection. Furthermore, it significantly prolongs the data conversion process from Lidar polar coordinates to the image format. Conversely, reducing the 2D image resolution results in a substantial loss of information. To illustrate this, consider a room measuring 6.00 × 4.00 m^2^. With a grid size of 60 × 40, each 0.10 × 0.10 m^2^ area is represented by a single pixel. However, with a grid size of 120 × 80, each 0.05 × 0.05 m^2^ area is depicted by a single pixel. The level of detail differs significantly, and given an average foot length of about 0.25 cm, only two to three pixels will represent the lower grid size when the Lidar is pointing directly from the left or right of the foot.

### 6.3. Classification Results—A Deeper Look

In this subsection, we aim to evaluate our proposed method in more detail for the different tasks before comparing it to the existing methods using the selected settings.

The neural network detailed in Section 4.3 was initially trained on the dataset tailored for task (T1). This neural network is denoted as “Net-1”. Subsequently, for the remaining tasks, the neural network weights, excluding the final fully-connected layer, are transferred and utilized in the two other networks employed for tasks (T2) and (T3) after fine-tuning. Accordingly, the neural networks assigned to tasks (T2) and (T3) are designated as “Net-2” and “Net-3,” respectively. “Net-2” and “Net-3” are initialized with the weights inherited from Net-1 and are further fine-tuned using their respective datasets.

In the following, we introduce the results of classification for the different tasks using these networks, when employing the previously found optimal settings.

#### 6.3.1. Activity Detection

As previously mentioned, “Net-1” undergoes training from the ground up using the data set employed for (T1). The neural network undergoes 500 epochs of training, utilizing the Adam optimizer with a batch size of 16 and a learning rate set to 0.001. Table 8 presents the accuracy, precision, recall, and F1 scores of the classification for task (T1). In addition, in Table 9, we show the confusion matrix of classification. It is evident that the most challenging classes to distinguish are “Fall” and “Lay Down,” with recall rates of 95.00% and 94.50%, respectively. This can be also seen from the confusion matrix, where instances of these two classes are the most misclassified with one another. The overall detection accuracy attains 96.10%.

#### 6.3.2. Fall Detection

For this task, all classes except for “Fall” are aggregated into a single one designated as “Non-Fall”. Subsequently, the network undergoes fine-tuning on the dataset previously outlined. Rather than training it from scratch, we start with the weights of the neural network trained for (T1). Other than the last dense layer, which is replaced by a new one composed of solely two neurons (for the two classes), the remaining layers start with these trained weights. The classification results for task (T2) are shown in Table 10. In addition, the confusion matrix of classification is given in Table 11. Notably, accuracy rates for the “Fall” and “Non-Fall” classes achieve 99.25% and 99.00%, respectively, culminating in an overall detection accuracy of 99.13%. It is, thus, possible to have high detection accuracy when the goal is to identify ”Fall” from ”Non-Fall” activities. This is intuitive as the higher the number of classes is, the more difficult it is for the neural network to distinguish between them. However, depending on the requirements of the system (i.e., whether a fine-grained HAR is required or a coarse-grained one), different neural networks could be invoked.

#### 6.3.3. Unsteady Gait Detection

In contrast to the prior two tasks, the objective of identifying an unsteady gait in this task is to anticipate potential hazardous events rather than merely identifying them. In this context, our goal is to recognize instances when an individual is walking with abnormalities. The neural network is fine-tuned using the data set described in Table 4. The classification results for task (T3) are shown in Table 12. In addition, the confusion matrix for this classification task is given in Table 13. The accuracy rates for the “Steady Gait” and “Unsteady Gait” classes achieve 95.25% and 91.13%, respectively, contributing to an overall detection accuracy of 93.13%.

It is clear that the detection of unsteady gait is harder than the detection of falls or even the general HAR. This is because the difference between patterns in this context is much larger. This can be seen more clearly when we check the detection accuracy for Lidar heights higher than 35 mm. For instance, when placed at heights equal to 200 mm and 250 mm, the accuracy reaches 65.7% and 63.9% respectively.

### 6.4. Proposed Approach against Conventional One

In this subsection, we conduct a comparative analysis between the results achieved by our proposed classification method and those of traditional methods introduced in [17,18]. It is worth noting that the approaches presented in these works heavily rely on a single Lidar and have predominantly been assessed in sparsely furnished rooms. In [18], despite tests conducted in various room environments, the activities were primarily performed in unobstructed areas. Similarly, in [17], the focus was on a single room setting, specifically a kitchen. While these methods rely solely on a single Lidar, our approach not only leverages the use of multiple Lidars but also formulates a more comprehensible data representation, enhancing the neural network’s classification accuracy. To faithfully reproduce the methodologies presented in [17,18], we implemented these approaches using individual Lidar data. During this comparison with conventional methods, we adapted the input format to align with the new data structure. For reference, in all rooms we designate the northernmost Lidar with index 1 and increment the index as we move clockwise to other Lidars. The classification results obtained using various Lidars when applying the method from [18] alongside our new method are presented in Table 14. Evidently, our proposed approach consistently surpasses the performance of the traditional method for all the Lidars utilized. This outcome is expected, given that our approach harnesses the complete information from all Lidars to construct a 2D map of objects within the room, akin to Simultaneous Localization And Mapping (SLAM).

As mentioned earlier, the comparison presented in Table 14 might seem as unfair since our proposed method is evaluated when using information from four different Lidars, while the conventional ones use a single Lidar at a time. Therefore, we conducted a final evaluation of the proposed method against the conventional ones, considering the combined data from all the Lidars. The data are transformed faithfully to fit within these methods for a fair comparison. In addition, a modified version of the network used in [38], which uses an inception module, as well as the approach proposed in [20], which relies on a moving Lidar having been tested; their results have been included for comparison. The results of classification for task (T1) are given in Table 15. As can be seen, even when including the data from all the Lidars at once, our proposed method outperforms the conventional ones by a large margin (i.e., over 10%), distinguishing it as the state-of-the-art for the task of activity detection using 2D Lidars.

### 6.5. Discussion

#### 6.5.1. Misclassification

Our method, despite outperforming the conventional ones, is not perfect. As the performance of classification reaches 96.1%, several instances have been misclassified. As shown in Table 9, the misclassified instances are spread among the different classes. However, it can be noted that the two classes “Fall” and “Lay Down” are the most confused classes. In particular, 14 instances of the class “Lay Down” were misclassified as being “Fall” activities, and 13 instances of the class “Fall” were misclassified the other way around. This is mainly due to the fact that these two activities are very similar in nature because the subject’s position changes from “standing” to “laying on the ground”. While differences can be observed in the 3D space (hands gesture, head orientation, etc.), such differences cannot always be detected by the 2D Lidars. A useful piece of information is the order in which the human body parts touch the ground because it serves as an important indicator that helps the LSTM capture the “Fall” activity. In the “Lay Down" activity, the hands usually precede the rest of the body, which is not necessarily the case for the “Fall” activity. Another observable difference resides in the time domain because fall activities tend to occur faster, and the change is sudden. Again, this is not necessarily always captured by the Lidars, especially when placed at a very low height as the changes occur in the 3D space, and the only observed events are ones that take place in the plane observed by the Lidars.

The next two confused activities are sitting down and standing, which both involve the points representing the feet stopping their movement. The difference between both resides in the placement in which this happens. When close to chairs or sofas, etc. they can be interpreted as sitting down, whereas if they occur far from such furniture, they are less likely to be interpreted as such.

Overall, our LSTM-based neural network manages to identify the activities quite effectively in most of the scenarios. The instances that have been misclassified can be correctly detected when given more training data to account for their nuances and to learn more specific patterns.

#### 6.5.2. Merits and Advantages

Unlike conventional methods, our proposed method is the first to employ sensor fusion to improve the accuracy of HAR and fall detection. On the one hand, single Lidars employed in works such as [17,18] have not been tested in environment with enough obstacles to validate to what extent they can perform these tasks (i.e., HAR and fall detection). In our work, as part of our simulations, we evaluated these methods in conditions where more obstacles have been introduced. As shown previously in Table 14 and Table 15, under such constraints, these conventional methods’ performance drops significantly. This is also the case even when multiple Lidars’ data are used. The idea of transforming the data into sequences of images, a part of our contribution in this work, allows as to make use of both the convolutional sub-network and the LSTM one to exploit both the spatial and temporal information in a way that identifies the different activities. Nonetheless, by identifying the optimal grid size in our early experiments (Section 6.2.3), the spatial information is selected to be as significant as possible. Larger grid sizes would make the points in the image sparse, hindering the convolutional sub-network from identification correlations between adjacent pixels. In a similar way, smaller grid sizes would make very few pixels include useful information, reducing the shape of the objects (namely, the human body) to a point where important features are missed. While the temporal information is accounted for in works such as [18,20,38] because they employ LSTM and time Inception modules, our method highly benefits from the 2D representation of the plane of detection, allowing for better performance.

#### 6.5.3. Limitations and Future Improvement

Throughout this work, we have demonstrated the usefulness of 2D Lidars are tools for human activity recognition and fall detection. The obtained results show a significant improvement over existing methods. That being said, these devices are not perfect. They come with technical and practical limitations that need to be addressed. In Table 1, we introduced two parameters that were meant to simulate real-world behavior. These parameters are the loss rate per rotation and the distance error. These parameters are set to [0–5%] and [0.5–2.0%], respectively. The selected values mean that for each Lidar, a random personage varying from 0.5% to 5% of the measurements is lost and that every measurement has an error ranging from 0.5% to 2.0%. For more realistic simulations, data need to be captured by a real Lidar, statistics regarding the data loss and measurement inaccuracies need to be calculated empirically, and the reported values need to be used in the simulations.

In addition to these limitations, other challenges need to be addressed. For instance, the commercially available Lidars do not offer diversity in terms of the light frequency/wavelength range in which they operate. In real-world deployment, this leads to high interference between the different Lidars, leading to inaccurate measurements, a high level of noisy data points, and overall unreliable detection. Sources of light spanning over the frequency ranges at which the Lidar operates, such as the sun light, can cause similar noise and interference. To address this problem, it is necessary to have access to Lidars operating at a configurable light bandwidth so that the different sensors operate with no risk of interference. However, operating at different frequencies could also mean different levels of noise sensitivities, which adds to the overall complexity of the system. This technical limitation is highly manufacturer-dependent and has not been addressed in our simulation.

Nevertheless, in real-world scenarios, mechanical devices (such as Lidars) are prone to failure. In our current work, we have assumed that the Lidars operate correctly at any given moment and that none of them has operational issues. Their collected and aggregated data give a decent representation of the status of the environment. However, if a Lidar or more goes/go down, the gathered measurements will lack major parts, leading to a significant drop in performance. This drop could reach results that are even lower than those reported for the conventional methods in Table 14 in the case of multiple failures. Such accuracy is unacceptable, and alternative solutions need to be provided. Such solutions include training different models for different failure scenarios and selecting the correct classification model depending on the failure nature.

## 7. Conclusions

In the context of this research paper, we introduced a novel approach for activity detection utilizing 2D Lidars. The contribution and novelty of this work involves the deployment of multiple Lidar devices within a single room. Through the concatenation of their sensed data, we demonstrated that it is possible to create more comprehensive representations of the activities performed. This, in turn, significantly enhances the accuracy of detection and enables precise fall detection. Diverging from our prior work described in [18], we adopted a fresh methodology. We generated a 2D image by concatenating the data sensed by the different Lidar scans and employed a ConvLSTM Neural Network for the classification task. Our approach was evaluated across three distinct tasks: activity detection, fall detection, and unsteady gait detection, and the achieved accuracy rates were 96.10%, 99.13%, and 93.13%, respectively. In future endeavors, we plan to conduct more extensive experiments to provide a more thorough assessment of our proposed method. Additionally, we aim to address the challenge of interference between the various Lidars, and the inaccuracies of distance estimation in realistic environments as well as the data loss problem, to further enhance the robustness and reliability of our approach.

## Figures and Tables

**Table 1:** The parameters of the 2D Lidar set in the simulator.

Parameter	Value
Scan rate	10–20 Hz
Measurements per rotation	720
Loss rate per rotation	0–5%
Distance range	25 m
Distance error	0.5–2.0%
Angular resolution	0.3375°

**Table 2:** The structure of the data set used for (T1).

Activity	Walk	Stand	Sit	Fall	Lay
Training set	500	500	500	500	500
Test set	400	400	400	400	400

**Table 3:** The structure of the data set used for (T2).

Activity	Fall	Non-Fall
Training set	500	500
Test set	400	400

**Table 4:** The structure of the data set used for (T3).

Activity	Steady Gait	Unsteady Gait
Training set	500	500
Test set	400	400

**Table 5:** The classification results of task (T1) for the early fusion vs. late fusion.

Activity	Accuracy	Prec.	Rec.	F1-Score
Early fusion	96.10%	96.10%	96.10%	96.10%
Late fusion	74.12%	74.12%	74.12%	74.12%

**Table 6:** The classification results of task (T1) for different heights of the Lidar.

Activity	Accuracy	Prec.	Rec.	F1-Score
height = 5 mm	96.32%	96.32%	96.32%	96.32%
height = 35 mm	96.10%	96.10%	96.10%	96.10%
height = 100 mm	93.88%	93.88%	93.88%	93.88%
height = 200 mm	88.14%	88.14%	88.14%	88.14%
height = 250 mm	81.67%	81.67%	81.67%	81.67%

**Table 7:** The classification results of task (T1) for different grid sizes.

Activity	Accuracy	Prec.	Rec.	F1-Score
60 × 40	89.15%	89.19%	89.15%	89.17%
90 × 60	96.10%	96.10%	96.10%	96.10%
120 × 90	92.85%	92.86%	92.85%	92.86%

**Table 8:** The classification results of task (T1).

Activity	Accuracy	Prec.	Rec.	F1-Score
Walk	97.75%	96.78%	97.75%	97.26%
Stand	97.00%	95.80%	97.00%	96.40%
Sit down	96.25%	97.22%	96.25%	96.73%
Fall	95.00%	95.00%	95.00%	95.00%
Lay Down	94.50%	95.70%	94.50%	95.09%
**Overall**	96.10%	96.10%	96.10%	96.10%

**Table 9:** The classification confusion matrix of task (T1).

Class	Classified as				
	Walk	Stand	Sit Down	Fall	Lay Down
Walk	**391**	5	0	1	3
Stand	5	**388**	6	0	1
Sit down	4	6	**385**	5	0
Fall	3	2	2	**380**	13
Lay Down	1	4	3	14	**378**

**Table 10:** The classification results of task (T2).

Activity	Accuracy	Prec.	Rec.	F1-Score
Fall	99.25%	99.00%	99.25%	99.13%
Non-Fall	99.00%	99.25%	99.00%	99.12%
**Overall**	99.13%	99.13%	99.13%	99.13%

**Table 11:** The classification confusion matrix of task (T2).

Class	Classified as	
	Fall	Non-Fall
Fall	**397**	3
Non-fall	4	**396**

**Table 12:** The classification results of the task (T3).

Activity	Accuracy	Prec.	Rec.	F1-Score
Steady	95.25%	91.37%	95.25%	93.27%
Unsteady	91.13%	93.20%	93.13%	93.12%
**Overall**	93.19%	92.28%	94.19%	93.20%

**Table 13:** The classification confusion matrix of task (T3).

Class	Classified as	
	Steady Gait	Unsteady Gait
Steady gait	**381**	19
Unsteady gait	36	**364**

**Table 14:** The classification results of the proposed method against the conventional ones [17,18] for task (T1).

Activity	Accuracy	Prec.	Rec.	F1-Score
Lidar 1 [18]	77.30%	77.39%	77.30%	77.34%
Lidar 2 [18]	78.85%	78.90%	78.85%	78.88%
Lidar 3 [18]	78.10%	78.10%	78.10%	78.10%
Lidar 4 [18]	80.30%	80.33%	80.30%	80.31%
Lidar 1 [17]	63.55%	63.50%	63.55%	63.53%
Lidar 2 [17]	65.11%	64.96%	65.11%	65.03%
Lidar 3 [17]	66.86%	66.80%	66.86%	66.83%
Lidar 4 [17]	68.21%	68.20%	68.21%	68.21%
Proposed	96.10%	96.10%	96.10%	96.10%

**Table 15:** The classification results of the proposed method against the conventional ones [17,18,20,38] for task (T1) using all the Lidars.

Activity	Accuracy	Prec.	Rec.	F1-Score
Luo et al. [17]	76.39%	76.39%	76.39%	76.39%
Bouazizi et al. [18]	84.88%	84.86%	84.88%	84.87%
Bouazizi et al. [20]	91.31%	91.31%	91.31%	91.31%
Roche et al. [38]	69.74%	69.74%	69.74%	69.74%
Proposed	96.10%	96.10%	96.10%	96.10%

## Data Availability

This work uses simulated data created using the simulation software introduced in ref. [20].

## Supplementary Materials

The following supporting information can be downloaded at: www.mdpi.com/xxx/s1, Video S1: A visualization of a simple simulated environment with simple obstacles, the moving robot and the fixed Lidars at the corners.; Video S2: The detection from the moving Lidar’s point of view. The 3 red layers indicate the different heights at which the rays are being cast and whose reflection is measured.

## Abbreviations

The following abbreviations are used in this manuscript:

AI	Artificial Intelligence
AMASS	Archive of Motion capture As Surface Shapes
ConvLSTM	Convolutional Long Short-Term Memory
CNN	Convolutional Neural Network
DALY	Disability-Adjusted Life Year
DBSCAN	Density-Based Spatial Clustering of Applications with Noise
DL	Deep Learning
EKF	Extended Kalman Filter
FN	False Negative
FP	False Positive
HAR	Human Activity Recognition
HOG	Histogram of Oriented Gradients
IR	Infrared
kNN	k Nearest Neighbors
Lidar	LIght Detection And Ranging
LOS	Line Of Sight
LRF	Laser Range Finder
LSTM	Long Short-Term Memory
RGB	Red Green Blue (color channels)
RGB-D	Red Green Blue Depth (color + Depth channels)
ROS	Robot Operating System
RNN	Recurrent Neural Network
SLAM	Simultaneous Localization and Mapping
SSD	Single Shot MultiBox Detector
SVM	Support Vector Machine
TCN	Temporal Convolutional Network
TN	True Negative
TP	True Positive
URDF	Unified Robot Description Format
XAI	eXplainable Artificial Intelligence
